# Early Effects of Neutering on Energy Expenditure in Adult Male Cats

**DOI:** 10.1371/journal.pone.0089557

**Published:** 2014-02-26

**Authors:** Alfreda Wei, Andrea J. Fascetti, Kyoungmi Kim, Ada Lee, James L. Graham, Peter J. Havel, Jon J. Ramsey

**Affiliations:** 1 Department of Molecular Biosciences, School of Veterinary Medicine, University of California Davis, Davis, California, United States of America; 2 Division of Biostatistics, Department of Public Health Sciences, School of Medicine, University of California Davis, Davis, California, United States of America; 3 Department of Nutrition, University of California Davis, Davis, California, United States of America; University of Colorado Medical School, United States of America

## Abstract

The initial cause of post-neutering weight gain in male cats is not entirely known. There is evidence that energy intake (EI) increases rapidly post-neutering, but it is not clear if neutering also decreases energy expenditure (EE) prior to weight gain. Thus, the purpose of this study was to determine if a decrease in EE contributes to the initial shift toward positive energy balance in neutered male cats. To determine the influence of neutering on EE independent of changes in EI and body weight (BW), male cats were fed at their pre-neutering maintenance EI and EE was measured at 4 days pre-neutering, 3–4 days post-neutering, and 9 days post- neutering. *Ad libitum* food access was then provided for 6 months. Body composition was measured and blood samples collected for serum chemistry at pre-neutering and 7 days, 13 days and 6 months post-neutering. Total energy expenditure (TEE) adjusted for lean body mass (LBM) did not change in cats from pre-neutering to 9 days post-neutering. However, TEE adjusted for BW and resting energy expenditure adjusted for either LBM or BW showed a small, but significant (P<0.05) increase from pre-neutering to 9 days post-neutering. When allowed free choice food access, cats showed significant increases of food intake (FI) and BW. Circulating concentrations of ghrelin increased, while adiponectin levels decreased following neutering. The results of this study indicate that initial post-neutering weight gain in male cats results from increased FI and not decreased EE. Long-term control of FI should be initiated after neutering to prevent hyperphagia and weight gain in male cats.

## Introduction

In the only large scale study of feline obesity prevalence in the United States, it was reported that 35% of adult cats were overweight or obese [1]. While this study used data from 1995, there is little reason to expect that the prevalence of feline obesity has decreased in recent years. The potential health consequences of obesity are significant since obesity in domestic cats is associated with diabetes mellitus, osteoarthritis, and other metabolic disorders [2], [3]. The development of successful strategies to prevent obesity is dependent on understanding the primary factors that induce weight gain in cats. Neutering is recognized as one of the major factors contributing to obesity in male cats, although the mechanisms that promote weight gain following this procedure are not well understood.

Weight gain resulting in obesity occurs when EI exceeds EE over a period of time, and an increase in FI [4], [5] and decrease in EE [4], [6], [7] have both been suggested as mechanisms contributing to post-neutering weight gain. Several studies have reported that gonadectomy stimulates increased FI in cats [4], [5], [8]. In a study reported by Kanchuk et al. [5], energy intake significantly increased within days of neutering in male cats, and weight gain began shortly thereafter. The influence of gonadectomy on EE and weight gain is less clear. It has been reported that following gonadectomy, EE decreases [7], does not change [5], [9], or either decreases or does not change depending on gender [4] or method used to normalize EE data for body size or composition [6]. It is difficult to make a strong conclusion from these studies about the role EE plays in initial weight gain with neutering because the studies were completed at different times after neutering and in all cases after significant increases in FI or BW had occurred. By this time, it is too late to determine if a change in EE caused the weight gain or if the change in EE is a consequence of the weight gain. Since Kanchuk et al. [5] showed that EI increases within the first week of neutering, it is essential to determine EE during this same time frame (before EI and BW increases) to determine if it plays any role in the initial shift toward positive energy balance following neutering. Therefore, in the present study, a 2 week post-neutering time frame was used for determinations of EE so that the cause of initial weight gain following neutering could be determined before clear changes in BW occurred.

Thus, the purpose of this study was to examine the effects of neutering on EE and weight gain in a group of adult male cats. Indirect calorimetry was used to measure EE in these cats 4 days prior to neutering, 3–4 days after neutering, and 9 days after neutering. In order to effectively analyze early changes in EE independent of increases in EI and BW, cats were maintained at their pre-neutering maintenance EI for the duration of the calorimetry measurements. The effect of *ad libitum* access to food on EI and weight gain following indirect calorimetry measurements was also examined from 13 days post-neutering to 6 months post-neutering in order to observe when BW would stabilize. It was hypothesized that EE would not change in the male cats with neutering, and that weight gain would be a result of increased EI.

## Materials and Methods

### Animals and Diet

Nine intact, specific-pathogen free adult male domestic shorthair cats (ages ranged from 2.5–3.6 years at the start of the study, mean ± SEM = 2.9±0.1 years, median age = 2.8 years) were individually housed for the duration of the study at the University of California, Davis. The facility maintained room temperatures between 18–24°C and had a 14 h light/10 h dark cycle. At the start of the study, the mean ± SEM of the BW of the cats was 5.2±0.3 kg. Cats consumed a dry maintenance diet that was routinely fed at the cat colony prior to the start of the study. This diet was formulated to meet the nutritional recommendations established by the Association of American Feed Control Officials Cat Food Nutrient Profiles for all life stages [10]. The nutrient composition of the diet on a dry matter basis was 38% protein, 32% carbohydrate, 17% fat, 9% ash, and 4% crude fiber. The calculated metabolizable energy for the diet was 16.19 kJ/g (or 3.87 kcal/g) on a dry matter basis. This diet provided 37% of energy from fat, 34% from protein, and 29% from carbohydrates. The experimental protocols were approved by the Institutional Animal Care and Use Committee at the University of California, Davis (Animal Welfare Assurance Number A3433-01) and complied with the recommendations of the National Research Council Guide for the Care and Use of Laboratory Animals.

### Study design

Baseline *ad libitum* daily FI was measured for all cats for at least 2 weeks in order to determine maintenance EI. Daily *ad libitum* FI was determined as the difference in the weight of excess food offered to each cat minus the weight of food left uneaten after 24 h. Following completion of these measurements, pre-neutering body composition measurements (LBM and fat mass (FM)) were performed using the deuterium oxide (D_2_O, 99.8 atom %D, Fisher Scientific Co, Pittsburgh, PA) isotopic dilution method [11]. Indirect calorimetry measurements for EE were performed starting 4 days prior to neutering, 3–4 days after neutering, and 9 days after neutering. Cats were acclimated to the calorimetry chamber for at least 3 consecutive days before each period of EE measurements. This period of acclimatization has been shown to be sufficient for EE measurements [12]. Acclimatization was also based on observations of a return to normal feeding and litterbox behaviors for each cat. At least 2 successive EE measurements (within 10% repeatability) were completed for each cat's pre- and post- neutering time-points. Cats were fed their maintenance EI from 4 days before neutering until 13 days post-neutering. On the thirteenth day post-neutering, body composition was measured for each cat so that cats had at least 1 day between the last calorimetry measurement and body composition measurements. Cats were then given *ad libitum* access to food for 6 months to observe when BW would stabilize. At the end of 6 months, LBM and FM for each cat were determined again. Blood collections for biochemical analyses were taken on day 7 post-neutering and at the same time that body composition measurements were completed (at pre-neutering, 13 days post-neutering, and at 6 months post-neutering). Cats were weighed once a week and individual FI was recorded daily for the duration of the study. Body condition scores (BCS) using a 9-point scale [13] were assessed at least once a month by 1 investigator (J. Ramsey). Since only one calorimetry chamber was available, cats were staggered in the experiment so that determinations for maintenance EI were performed on at least one cat while another was undergoing calorimetry measurements.

### Neutering

Cats were fasted for 12 h (water was available *ad libitum*) prior to neutering by a standard open technique [14]. Cats received 0.3 mg/kg butorphanol (Fort Dodge Animal Health, Fort Dodge, IA), 0.05 mg/kg acepromazine maleate (Vedco, Inc., St. Joseph, MO), and 0.02 mg/kg atropine sulfate (Vedco, Inc., St. Joseph, MO) subcutaneously 30 min before induction. Cats were then induced with 2–3 mg/kg ketamine (Vedco, Inc., St. Joseph, MO) and 0.25 mg/kg diazepam (Hospira, Inc., Lake Forest, IL) intravenously. Additional isoflurane (Vet One, India) delivered by inhalation was used if needed. A subcutaneous dose of 1.5 mg/kg carprofen (Pfizer, Inc., Madison, NJ) was given post operatively and repeated 24 h later.

### Indirect respiration calorimetry

Indirect respiration calorimetry measurements were conducted on the male cats using a previously described method and calorimetry system [12]. Data collected for 12 h was extrapolated to 24 h since TEE and respiratory quotient (RQ) data obtained from 12 h and 24 h runs did not differ between day and night [12]. This is consistent with previous reports that cats do not have clearly defined circadian rhythms [15]. A data acquisition system (Labview, National Instruments, Austin, TX) interfaced to a computer collected data from a mass flowmeter (Flowkit 100, Sable Systems, Las Vegas, NV), O_2_ analyzer (AEI Technologies, Pittsburgh, PA) and CO_2_ analyzer (AEI Technologies, Pittsburgh, PA). The analyzers were calibrated with N_2_ gas, 1.90% CO_2_ standard gas, and dry outside air before each calorimetry run. Weekly calibrations of the entire calorimetry system were performed using an ethyl alcohol recovery. Energy expenditure was calculated using the Weir equation [16]. Resting EE was determined from stable periods of at least 30 min in duration where minimal spikes in EE were observed in plots of EE against time.

### Determination of body composition

Body composition measurements were determined before neutering, 13 days post-neutering, and 6 months post-neutering. Body FM and LBM was estimated by the D_2_O isotopic dilution method of Backus et al. [11] with modifications. The cats fasted for 12 h prior to body composition measurements. Water was taken away from cats 2 h before collection of a basal (without D_2_O enrichment) blood sample. Blood samples were collected by jugular venipuncture into additive-free tubes (Vacutainer, Becton-Dickinson Ltd., Franklin Lakes, NJ) and then allowed to clot for 30 min at 25°C. Deuterium oxide (0.4 g D_2_O/kg body weight) was then administered to the cats subcutaneously and allowed to equilibrate for 3 h. A blood sample (D_2_O enriched) was then collected again by jugular venipuncture for body composition measurements. All tubes were centrifuged for 10 min at 2,817 X *g* for serum harvest. Serum collected (from samples without D_2_O enrichment and D_2_O enriched samples) was then stored at −20°C until D_2_O extraction. Briefly, sample tubes each containing 75 µL of serum un-enriched with D_2_O were prepared in triplicate and placed inverted in a heated glass bead bath (48°C) for 24–48 h to ensure full condensation of water from serum. This was also repeated for D_2_O enriched serum. Approximately 50–60 µL of serum water would condense on the sides and tip of the inverted tube after complete condensation. Condensed serum water samples (30 µL) were then analyzed by a Fourier transform infrared spectrometer equipped with a class 2A laser (ATI Mattson Infinity Series, Madison, WI).

### Biochemical determinations

Whole blood samples were collected for each cat at pre-neutering, 7 days post-neutering, 13 days post-neutering, and 6 months post-neutering. Serum was collected from whole blood following separation by centrifugation. Serum samples were then analyzed for glucose, insulin, leptin, free fatty acids, triglycerides, adiponectin, and ghrelin. Analyses of blood metabolites and hormones were completed according to the directions of the commercially available analytical kits. Glucose and triglyceride concentrations were determined by enzymatic colorimetric assays (Fisher Diagnostics, Middletown, VA). Insulin, leptin and ghrelin were analyzed using a porcine insulin radioimmunoassay (RIA) kit, multi-species leptin RIA kit, and human total ghrelin RIA kit respectively, from EMD Millipore (St. Charles, MO). Concentrations of free fatty acids were determined by enzymatic colorimetric assay (Wako Diagnostics, Richmond, VA). Adiponectin concentrations were determined using a rat/mouse ELISA kit from B-Bridge (Cupertino, CA).

### Statistical Analysis

Prior to statistical analysis, normality and homoscedasticity of each variable were assessed with a Shapiro-Wilks test and Flinger-Killeen test, respectively. Statistical analysis was performed using R 2.12.0 language and environment (http://www.r-project.org). Paired student *t*-tests were used to determine the significance of mean differences between 2 time points for EE (i.e., TEE, TEE/metabolic BW (BW^0.75^), resting EE (REE), REE/BW^0.75^, RQ) and body composition variables as well as metabolic biomarkers. Multiple comparisons were controlled by the Tukey's HSD (Honestly Significant Difference) method. A two-sided p-value of 0.05 or less was considered significant. Energy expenditure measurements were adjusted with BW (at time of D_2_O measurements) and LBM to account for differences in individual body size and composition, respectively. Analyses of covariance (ANCOVA) were used to determine if differences in TEE adjusted for BW at time of D_2_O measurement (BW (D_2_O)) (denoted as “adjusted TEE^†^”) or LBM (denoted as “adjusted TEE^‡^”) existed between pre-neutering, 3–4 days post-neutering, and 9 days post-neutering. Differences in REE adjusted for BW (D_2_O) (denoted as “adjusted REE^†^”) or LBM (denoted as “adjusted REE^‡^”) were also determined between these time points using ANCOVA. Both pre-neutering TEE and REE were adjusted by using BW (D_2_O) or LBM from pre-neutering body composition measurements as covariates. Total EE and REE determined at 3–4 days post-neutering and 9 days post-neutering were adjusted using BW (D_2_O) or LBM from day 13 post-neutering body composition measurements as covariates. All data are reported as mean ± SEM unless specified otherwise, for consistency.

## Results

### Energy expenditure

There was no evidence to support the hypothesis that a decrease in EE may contribute to initial weight gain following neutering in male cats (Table 1). In fact, TEE increased (p<0.05) at both 3–4 and 9–10 days post-neutering when compared to pre-neutering. Similarly, REE increased (p<0.05) at 3–4 days post-neutering and showed a trend (p = 0.06) towards an increase at 9–10 days post-neutering compared to pre-neutering. However, when TEE and REE were adjusted for LBM or BW using ANCOVA there were no significant differences (p>0.10) between pre-neutering and 3–4 days post-neutering. TEE adjusted for LBM was also not different (p>0.10) between pre- and 9–10 days post-neutering. In contrast, TEE adjusted for BW and REE adjusted for either BW or LBM was increased (p<0.05) at 9-10 days post-neutering versus pre-neutering. TEE and REE divided by BW (kg)^0.75^ was increased at both 3–4 and 9–10 days post-neutering when compared to pre-neutering. Respiratory quotients did not differ between pre-and post-neutering.

**Table 1:** Food intake, body weight, and energy expenditure measurements in cats at pre-neutering, 3–4 days post-neutering, and 9-days post neutering.*

	Pre-neutering (days -4, -3)	Post-neutering (days 3, 4)	Post-neutering (days 9, 10)
**Characteristics** §			
FI (g/d)	62±4^a^	67±4^a,b^	70±6^b^
EI (kJ/d)	1006±65^a^	1078±61^a,b^	1136±98^b^
**Energy expenditures**			
BW (kg)	5.2±0.3^a^	5.2±0.3^b^	5.1±0.3^b^
RQ	0.84±0.02	0.85±0.02	0.85±0.02
TEE (kJ/d)	1108±62^a^	1174±71^b^	1177±74^b^
TEE/BW^0.75^ (kJ/kg/d)	321±11^a^	342±11^b^	344±10^b^
REE (kJ/d)	1030±66^a^	1103±68^b^	1090±77^a,b^
REE/BW^0.75^ (kJ/kg/d)	298±12^a^	321±10^b^	318±11^b^
Adjusted TEE† (kJ/d)	214±8^a^	230±6^a,b^	231±5^b^
Adjusted REE† (kJ/d)	199±8^a^	216±6^a,b^	213±5^b^
Adjusted TEE‡ (kJ/d)	253±10	267±6	268±7
Adjusted REE‡ (kJ/d)	235±11^a^	251±6^a,b^	248±8^b^

* Values are given as mean ± SEM. For rows in which superscripts are present, values in a row that do not share a common superscript differ by p<0.05.

† BW (D_2_O) included as a covariate.

‡ LBM included as a covariate.

§ FI  =  Food intake. EI  =  Energy intake. BW  =  Body weight. RQ  =  Respiratory quotient. TEE  =  Total energy expenditure. TEE/BW^0.75^  =  Total energy expenditure/Metabolic body weight. REE  =  Resting energy expenditure. REE/BW^0.75^  =  Resting energy expenditure/Metabolic body weight. LBM  =  Lean body mass.

The male cats showed a small, but significant (p = 0.02) decrease of BW between pre- and post-neutering calorimetry measurements (Table 1). This likely reflected the fact that they were food deprived prior to neutering, and EI was low on the day of neutering. All cats were fed their pre-neutering maintenance EI for the calorimetry measurements, although there was a significant (p = 0.03) difference in EI between the pre-neutering and 9–10 day post-neutering measurements. This likely reflected normal daily fluctuation in FI and neutering may have increased the drive for the cats to more consistently eat all food offered to them. Cats may have also increased FI at 9–10 days post-neutering in response to an augmented hunger drive that followed from being fasted twice in one week (for the neutering procedure and for the blood collection procedure on day 7 post-neutering).

### Energy Intake, BW, body composition, and BCS

**Figure 1:**
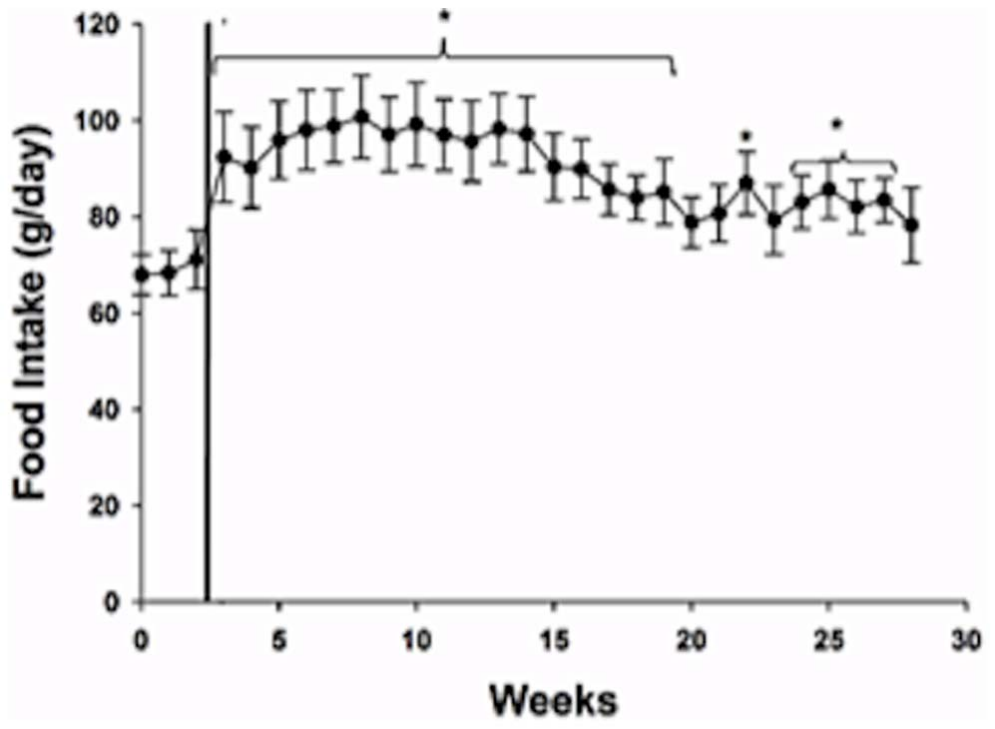
Mean (± SEM) weekly food intake (FI) in the male cats following neutering. Week 0 is designated as baseline (immediately before neutering). Cats were fed at pre-neutering maintenance FI from week 0 to week 2. The vertical line denotes the beginning of *ad libitum* feeding (week 3 to week 28). Asterisks over the brackets covering week 3 to week 19, and week 24 to week 27 denote significant differences in average weekly FI from pre-neutering maintenance FI, p<0.05.

Neutering in the male cats induced a period of hyperphagia (Figure 1). In Figure 1, week 0 was designated as baseline, and the cats were fed at pre-neutering maintenance EI for the first 2 weeks following neutering (weeks 1 and 2). From week 3 onward, the cats were allowed *ad libitum* access to food. As expected, there were no differences (p>0.10) in EI between weeks 0, 1, and 3 when cats were fed at pre-neutering maintenance EI. The cats showed an immediate (week 3) increase (p<0.05) in EI when they were given *ad libitum* food access, and this increase (p<0.05) above baseline continued except during weeks 20, 21, 23 and 28 (Figure 1).

**Figure 2:**
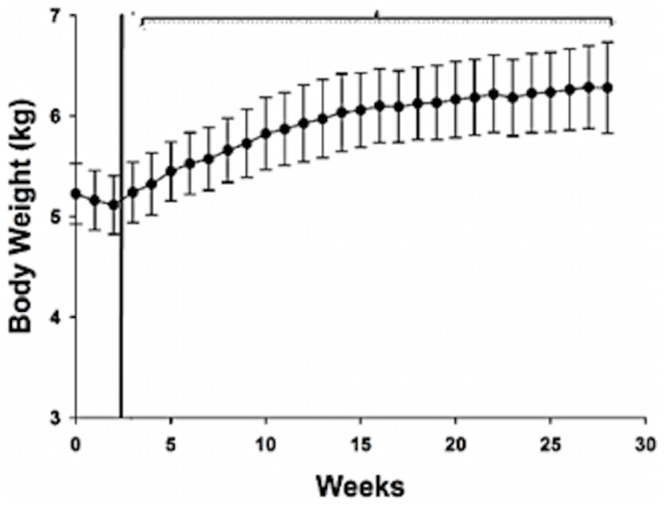
Mean (± SEM) weekly body weight (BW) in the male cats following neutering. Week 0 is designated as baseline (immediately before neutering). Cats were fed at pre-neutering maintenance food intake from week 0 to week 2. The vertical line denotes the beginning of *ad libitum* feeding (week 3 to week 28). The asterisk over the bracket covering week 4 to week 28 denotes significant differences in average weekly BW from the averaged values from week 0 to week 2, p<0.05.

The influence of neutering on BW and composition in male cats is summarized in Table 2 and Figure 2. There was no significant difference in BW, LBM and FM between pre-neutering and post-neutering day 13 (the last day cats were fed at pre-neutering maintenance EI). At 6 months post-neutering, BW and FM were significantly increased (p<0.05), and LBM showed a trend (p<0.10) towards an increase, compared to either pre-neutering or post-neutering day 13. Following 2 weeks of *ad libitum* food access (week 4 in Figure 2), cats showed a significant (p<0.05) increase in average weekly BW compared to baseline and this increase in BW continued for the duration of the study. Body condition scores for cats also significantly (p = 0.03) increased from 13 days post-neutering (BCS = 4.8±0.1) to 6 months post-neutering (BCS = 6.3±0.2).

**Table 2:** Body composition measurements determined in cats at pre-neutering, 13 days post-neutering, and 6 months post-neutering.*

	Pre-neutering	Post-neutering (day 13)	Post-neutering (6 months)
**Body composition** †			
BW (D_2_O) (kg)	5.2±0.3^a^	5.1±0.3^a^	6.3±0.4^b^
LBM (kg)	4.4±0.3	4.4±0.2	4.8±0.3
FM (kg)	0.8±0.1^a^	0.7±0.1^a^	1.4±0.3^b^
LBM (% BW)	85±1.4	86±2.4	78±4
FM (% BW)	15±1.4	14±2.4	22±4

* Values are given as mean ± SEM. For rows in which superscripts are present, values in a row that do not share a common superscript differ by p<0.05.

† BW (D_2_O)  =  Body weight at time of deuterium oxide (D_2_O) measurements. LBM  =  Lean body mass. FM  =  Fat mass. BW  =  Body weight.

### Serum chemistry

No significant differences in insulin and free fatty acid concentrations were observed in the male cats between any time points (Table 3). Significant (p = 0.01) increases of serum ghrelin concentrations were observed in cats at 7 days post-neutering and at 6 months post-neutering (p = 0.02) compared to pre-neutering. There was a significant (p = 0.04) increase of circulating leptin concentration and a significant (p = 0.04) decrease of glucose concentration at 6 months post-neutering compared to pre-neutering. As expected, serum leptin increased in conjunction with gains in weight and fat mass at 6 months post-neutering [17]. Triglyceride concentrations significantly (p<0.05) increased at 6 months post-neutering compared with either day 7 or 13 post-neutering. Adiponectin concentrations significantly (p<0.01) decreased from pre-neutering to day 7, day 13 or 6 months post-neutering (p<0.01).

**Table 3:** Serum chemistry results determined in cats at pre-neutering, 7 days post-neutering, 13-days post-neutering, and 6 months post-neutering.*

	Pre-neutering	Post-neutering (day 7)	Post-neutering (day 13)	Post-neutering (6 months)
**Serum chemistry**				
Glucose (mg/dL)	89.1±9.5^a^	77.3±3.6^a,b^	80.1±6.9^a,b^	72.0±3.4^b^
Insulin (μU/mL)	10.7±1.3	10.6±1.7	10.8±1.5	12.0±2.3
Leptin (ng/mL)	3.9±0.3^a,b^	3.6±0.3^a^	4.0±0.3^b^	6.3±1.0^c^
Free fatty acids (mEq/L)	0.36±0.06	0.37±0.06	0.35±0.04	0.38±0.05
Triglycerides (mg/dL)	32.7±6.3^a,c^	26.1±5.0^a,b^	24.4±3.7^b^	42.8±5.8^c^
Ghrelin (pg/mL)	727.3±54.2^a^	788.8±60.3^b,c^	767.1±35.8^a,b^	855.35±42.4^c^
Adiponectin (μg/mL)	7.8±1.3^a^	5.1±0.9^b^	5.2±0.7^b^	2.6±0.7^c^

*Values are given as mean ± SEM. For rows in which superscripts are present, values in a row that do not share a common superscript differ by p<0.05.

## Discussion

Decreased EE [6], [7], increased EI [4], [5], or a combination of the two [4] have been reported as factors contributing to post-gonadectomy weight gain in cats. However, there has not been agreement among previous studies about which factor or factors, are the largest contributors to initial weight gain following gonadectomy. There are two major reasons why it is difficult to make strong conclusions from these studies about the influence of EE on initial weight gain with gonadectomy. First, EE was measured in previous studies [4]–[7] after significant increases in BW and/or FI had already occurred. In the case of studies that reported a decrease in EE, EE was measured at least 5 months [7] or 6 months [6] after gonadectomy and well after initial changes in weight would have occurred. Thus, it is difficult to determine if changes in EE are a cause or a consequence of changes in BW. Also, most EE studies have been completed in *ad libitum* fed cats and increased FI following gonadectomy [4], [5] could influence EE. Second, it has been common to adjust EE for possible group differences in BW or composition by dividing EE by BW [5], [6], BW^0.75^ [4]–[6], or LBM [6]. Concern has been raised however, about the statistical validity of using ratios to normalize EE data [18], [19]. There is a growing consensus that regression approaches, such as ANCOVA, are generally the most appropriate way to adjust EE data for BW or composition [19], [20].

In the present study, the influence of neutering in male cats on EE, independent of increases in FI, was studied. Cats were fed at pre-neutering EI levels for the first 2 weeks following neutering. Determinations of EE during this time frame showed that EE did not shift energy balance toward the positive in male cats post-neutering. The results of the study indicate that neutering does not induce a decrease in EE at a time when previous studies have shown an increase in EI [5]. To normalize EE for BW and composition, EE was compared between time points using ANCOVA where BW or LBM were the covariates. There was no difference in mass-adjusted REE or TEE between pre-neutering and 3–4 days post-neutering, while REE (adjusted for either LBM or BW) and TEE (adjusted for BW) increased at 9–10 days post-neutering compared to pre-neutering. The reason for this increase in EE is not entirely clear, although it is not due to an increase in major movement since all animals were very sedentary during the calorimetry measurements as shown by TEE values that were only slightly larger than REE values. Also, only increases in REE and not TEE were observed for LBM adjusted EE data. It can be argued that the neutering surgery in male cats could influence EE, although there was no evidence to suggest that tissue injury stimulated EE, since significant increases in REE adjusted for either BW or LBM did not occur in the first 3–4 days post-neutering. It is possible that increased hunger may have caused fidgeting or small muscle movements in the male cats that would have been included with REE at 9–10 days post-neutering, although additional studies would be necessary to determine if this is true. Nonetheless, the results of this study clearly show that total EE or EE normalized for BW or composition is not decreased by neutering in male cats and thus cannot contribute to weight gain immediately following neutering. The positive energy balance (weight gain) that develops immediately post-neutering in male cats is thus clearly driven by increased energy intake.

To determine the influence of neutering on FI, the cats were allowed continuous access to food from 13 days post-neutering until the end of the study. It has been previously reported that an increase in FI occurs by 3 days after gonadectomy in male cats [5], and an increase in FI has been reported to be a major factor contributing to weight gain in gonadectomized cats [4]. The results of the present study support this conclusion and indicate that hyperphagia following neutering is the cause of initial weight gain in male cats. It has previously been shown that FI is increased for at least 3 months after neutering in male cats when compared to pre-neutering values [4]. The present study indicates that neutering in male cats produces an increase in FI above pre-neutering values for as long as 27 weeks. Therefore, long-term control of FI needs to be initiated after neutering to prevent hyperphagia and weight gain in male cats.

In the present study, the male cats showed a 20% increase in BW from pre-neutering to 6 months post-neutering. This was comparable to the ∼28% increases in BW exhibited by neutered normal and neutered lipoprotein lipase-deficient male cats fed *ad libitum* for 9 months post-neutering in the Kanchuk et al. study [5]. Body composition analyses showed that weight gain observed in the male cats in the present study resulted mainly from a mean increase in FM of 75% from pre-neutering to 6 months post-neutering. In contrast, there was no significant increase in LBM from pre-neutering to 6 months post-neutering. The magnitude of the increase in FM was consistent with results from previous studies that measured body composition 6–9 months after neutering in male cats [5], [9]. These results support the idea that neutering in adult male cats causes an increase in BW primarily through an increase in FM.

In the present study, serum metabolite and hormone levels were measured pre- and post-neutering. Weight gain while cats had *ad libitum* food access did not significantly affect fasting serum glucose, insulin, or free fatty acid concentrations. However, increased serum concentrations of leptin and triglycerides accompanied the increases in FM and BW, and were consistent with other reports [4]–[6], [11], [21]. Adiponectin is an adipokine involved in insulin sensitization, modulation of energy and lipid metabolism, and inflammation [22]–[24]. Cats exhibited a decreased serum adiponectin concentration following neutering and after 6 months of weight gain promoted by free choice food access. These results are consistent with previous reports that have shown decreased adiponectin concentrations in overweight and obese cats compared with normal (based on BCS ratings) healthy cats [22]–[24]. In humans that underwent weight loss, increased adiponectin concentrations were also negatively correlated to changes in BW, FM and body mass index [25]. For the present study, it is also notable that no previous studies in cats have measured adiponectin concentrations at so early a time-point following gonadectomy, and thus these studies have provided no information about how quickly adiponectin concentrations change in response to neutering within male cats. The results of the present study indicate that neutering of male cats causes a rapid decrease in adiponectin concentrations that occurs before changes in BW are observed. In contrast to the studies in cats, however, there are published studies in rodents and humans reporting that castration or hypogonadism is associated with increased adiponectin and that testosterone administration reduces circulating adiponectin concentrations [25]. The reason for this difference between cats and other species remains to be determined. For both humans and rodents, adiponectin concentrations are sexually dimorphic (with males having lower concentrations), with the inhibition of adiponectin production during puberty being mediated by testosterone [25]. Interestingly, the serum levels of ghrelin, a peptide hormone that plays a role in the regulation of FI, BW, and energy homeostasis [26], increased following neutering and remained elevated through 6 months post-neutering. Several studies have shown that ghrelin stimulates FI in laboratory rodents [27]–[29] and humans [30], [31]. However, little is known about the influence of neutering on ghrelin in male cats. The results of the present study suggest that ghrelin should be considered as a factor contributing to increases of FI following neutering in male cats.

There are a couple of possible limitations to the present study that need to be considered. First, a control group of intact male cats was not included. Second, the study does not determine the long-term effects of neutering on EE in male cats. It is possible that changes in BW and long-term alterations in hormone levels could eventually lead to alterations in mass-adjusted EE in neutered male cats. However, the present study clearly shows that a decrease in EE does not contribute to initial weight gain following neutering in male cats. Third, the study was completed only in males and the conclusions of this study should not be extrapolated to spayed females. In fact, there is some evidence to suggest that females could show a different energetic response to gonadectomy than males. It has been reported that maintenance energy requirements are decreased following spaying in female cats [8], [32], [33] while no significant change is observed in neutered male cats [32]. In conclusion, the results of this study indicate that a decrease in EE does not contribute to weight gain following neutering in male cats. Weight gain in the male cats occurred as a consequence of increased FI, which could be partially due to elevated serum ghrelin levels. Long-term control of FI needs to be initiated after neutering to prevent hyperphagia and weight gain in male cats.
